# Comparing bacterial dynamics for the conversion of organics and humus components during manure composting from different sources

**DOI:** 10.3389/fmicb.2023.1281633

**Published:** 2023-09-28

**Authors:** Yan Li, Jun Li, Yuan Chang, Ruoqi Li, Kaiyun Zhou, Yabin Zhan, Renyue Wei, Yuquan Wei

**Affiliations:** ^1^Central Laboratory, Haikou Affiliated Hospital of Central South University Xiangya School of Medicine, Haikou, China; ^2^Haikou City Key Laboratory of Clinical Medicine, Haikou, China; ^3^Key Laboratory of Tropical Biological Resources, Ministry of Education, Key Laboratory for Marine Drugs of Haikou, Hainan University, Haikou, China; ^4^College of Resources and Environmental Science, Beijing Key Laboratory of Biodiversity and Organic Farming, China Agricultural University, Beijing, China; ^5^Organic Recycling Institute (Suzhou) of China Agricultural University, Suzhou, China; ^6^Key Laboratory of Fertilization from Agricultural Wastes, Ministry of Agriculture and Rural Affairs, Institute of Plant Protection and Soil Fertilizer, Hubei Academy of Agricultural Sciences, Wuhan, Hubei, China; ^7^College of Life Science, Northeast Agricultural University, Harbin, China

**Keywords:** manure composting, organic components degradation, humification index, bacterial community, humus carbon and nitrogen

## Abstract

The study aimed to compare the differences in organic fractions transformation, humus components and bacterial community dynamics during manure composting from different sources, and to identify the key biotic and abiotic factors driving the humification process. Five types of manure [pig manure (PM), cow dung (CD), sheep manure (SM), chicken manure (CM), and duck manure (DM)] were used as raw materials for 30 days composting. The results showed the obvious difference of organic fractions decomposition with more cellulose degradation in CD and SM composting and more hemicellulose degradation in PM and CM composting. Composting of PM and CD contained significantly higher humus fractions than the other composts. Fluorescence spectra indicated that SM composting tended to form structurally stable humic acid fractions, while CM and DM tended to form structurally complex fulvic acid fractions. Pearson correlation analysis showed that humification process of composts in category A (PM, CD) with higher humification degree than category B (SM, CM, and DM) was positively correlated with lignin and hemicellulose degradation. Bioinformatics analysis found that *Lysinibacillus* promoted the degradation of hemicellulose and the conversion of fulvic to humic acid in the composts of category A, and in category B, *Thermobifida*, *Lactobacillus*, and *Ureibacillus* were key genera for humic acid formation. Network analysis indicated that bacterial interaction patterns had obvious differences in composting with different humus and humification levels.

## 1. Introduction

With the development of economy in China and the increasing demand for meat, the livestock and poultry farming industry is developing rapidly, and the accompanying environmental pollution from livestock and poultry manure is becoming increasingly serious (Sun et al., 2012). Untreated animal manure generally contains many pathogenic bacteria, parasitic eggs, and breeding flies, which may cause infectious diseases of human and animal. On the other hand, it is estimated that 3.8 billion tons of livestock and poultry manure are produced per year in China, which can provide about 53 million tons of nitrogen, phosphorus and potassium, accounting for 89% of the total amount of chemical fertilizer applied in China (Zhang et al., 2023). Therefore, recycling the nutrients from manure into the soil is a crucial way to replace chemical fertilizer, which is beneficial for the green development of agriculture.

Aerobic composting is a biochemical process mediated by microbes and a way of resource utilization and harmless disposal of livestock and poultry manure, which produces a mature compost fertilizer or a soil conditioner based on the transformation of organic matter (e.g., cellulose and lignin, etc.) to humus (Wang et al., 2021; Wei et al., 2022). Humus as the stable organic matter is important for the quality of compost products, which mainly includes fluvic acid (FA), humic acid (HA) and humin (Schellekens et al., 2017; Xu et al., 2022). Many studies have shown that the decomposition of lignocellulose produced polyphenols, quinones, polysaccharides or reducing sugars, and these organic intermediates could be directly utilized by microorganisms or participate in humus formation (Wang et al., 2022). It is reported that the conversion and humification of organic components from diverse raw materials are quite complex and different (Gao et al., 2015), and most of the researches focused on the composting using a single kind of manure as raw material (Ma et al., 2022; Zhang et al., 2022). The comparative research for the composting from different livestock and poultry manure is rarely reported. In-depth research on understanding the diverse forming regular of humus in manure composting from different sources will be beneficial for optimizing raw material formula and improving the quality of composts.

The humification process is closely related to the dynamic action of complex microbial community in composting (Albrecht et al., 2010). Studies have shown that Proteobacteria, Firmicutes, Actinobacteria, and Bacteroidetes are the dominant phyla in a variety of manure composting processes, which may contribute to humus formation and organics stabilization (Zhang et al., 2021). It is reported that most of the microorganisms involved in polysaccharide hydrolysis and lignocellulose degradation could provide the main precursors for humus formation (Wang et al., 2023). Humus formation is affected by multiple metabolisms of microbial communities, such as citrate-cycle, pentose phosphate pathway, pyruvate metabolism, etc. (Yang et al., 2023) and potentially regulated by microbial community succession and interaction (Zhan et al., 2023). In recent years, network analysis among microbial species and physicochemical properties has become an important approach to determine the role of microorganisms in composting (Yu et al., 2023), which is widely used to understand the interaction of microbial community, the release of metabolites, cellulose degradation, and humus synthesis (Zheng et al., 2021). However, few reports were about the relationship between bacterial communities, organics conversion, and humification in diverse manure composting.

The aim of this study was to: (1) investigate the organic fractions conversion patterns in manure composting from five different sources, (2) assess the changes in humus fractions of different manure composts, (3) compare the dynamics of bacterial community composition and interaction, and (4) identify the correlations between humification indicators and key microorganisms in different manure composts. This study will provide a theoretical basis for further optimizing organics conversion and improving humification efficiency for manure composting.

## 2. Materials and methods

### 2.1. Composting experimental design

Five kinds of manure were prepared for composting as raw materials, including pig manure, cow manure, sheep manure, chicken manure and duck manure. Pig manure and cow manure were collected from a farm in Huzhou City, Zhejiang Province; sheep manure was taken from a sheep breeding center in Suzhou City, Jiangsu Province; chicken manure and duck manure were taken from a poultry farm in Jiangsu Province. Sawdust was used to regulate the ratio of carbon to nitrogen (C/N), which was purchased from a wood processing plant in Suzhou City. The basic characteristics of raw materials were provided in Supplementary Table S1. Pig manure, cow dung, sheep manure, chicken manure and duck manure were mixed with sawdust at a ratio of 4:1 (m:m, fresh weight) for composting, and the five treatments were recorded as PM, CD, SM, CM, and DM, respectively. Composting with 3 repetitions for each treatment was performed for 30 days in a 10 L reactor as described by Zhan et al. (2021) and ~ 8 kg fresh weight of raw material was composted in the reactor. The aeration rate was set at 0.2 L kg·DM^−1^·min^−1^ (aeration 30 min and stop 30 min), and deionized water was added during composting to maintain the moisture of the piles at about 60%. The samples (about 100 g) were collected on day 0, 4, 10, 20, and 30, and were stored at −20°C.

### 2.2. Analysis for physicochemical properties

Temperatures data were collected by a digital thermometer each day. Germination Index (GI), pH, and electrical conductivity (EC) were measured according to China’s standard for organic fertilizer (NY/T 525-2021). Total organic carbon (TOC) and total nitrogen (TN) were determined by an elemental analyzer (Elementar vario MACRO cube, Germany). The contents of ammonium nitrogen and nitrate nitrogen were determined by a continuous flow analyzer. The content of lignocellulose fractions (cellulose, hemicellulose, and lignin) was determined according to Van Soest et al. (1991).

### 2.3. Extraction and determination of humus fractions

Humus fractions were isolated and extracted as described by Zhao et al. (2020), which were divided into humic acid (HA) and fulvic acid (FA). The carbon and nitrogen content of HA and FA were determined by an elemental analyzer (Elementar vario MACRO cube, Germany). Three-dimensional fluorescence spectra of HA and FA were analyzed with a fluorescence spectrophotometer (F-7000, Hitachi Japan). The excitation-emission matrix (EEM) fluorescence data were analyzed according to Wei et al. (2007) and the interference of Rayleigh and Raman scattering was removed according to Bahram et al. (2006).

### 2.4. Bacterial community analysis

The extraction of DNA from compost samples was conducted based on the FastDNA spin Kit for soil (MP, Biomedicals, Santa Ana, Carlsbad, CA, United States). Primers for 16S rRNA gene amplification were the universal primers 515F (5′-GTGCCAGCMGC CGCGGTAA-3′) and 909R (5′-CCCCGYCAATTCMTTTRAGT-3′) fused with a 12 nt unique barcode (Wang et al., 2020). Purified PCR products were sequenced using the Illumina Hiseq platform. Low-quality sequences were removed and OTUs were classified by QIIME as previously described by Zhan et al. (2021). The sequences were submitted to the NCBI (PRJNA730304). The “Vegan” package in R 3.1.2 was used to compare the microbial composition based on the Bray-Curtis distance and alpha diversity index (Chao1 index, Shannon index) was calculated according to Wang et al. (2021).

### 2.5. Statistical analysis

IBM SPSS (Version 22.0, United States) was used to analyze physicochemical data for estimating the statistical significance at a confidence level of *p* < 0.05 and the data was shown as the means of triplicates based on Origin (Version 2021, United States) software. Gephi 0.9.2 was used to visualize bacterial correlation networks at genus level.

## 3. Results and discussion

### 3.1. Changes of physicochemical properties and maturity indexes of manure compost from different sources

**Figure 1:**
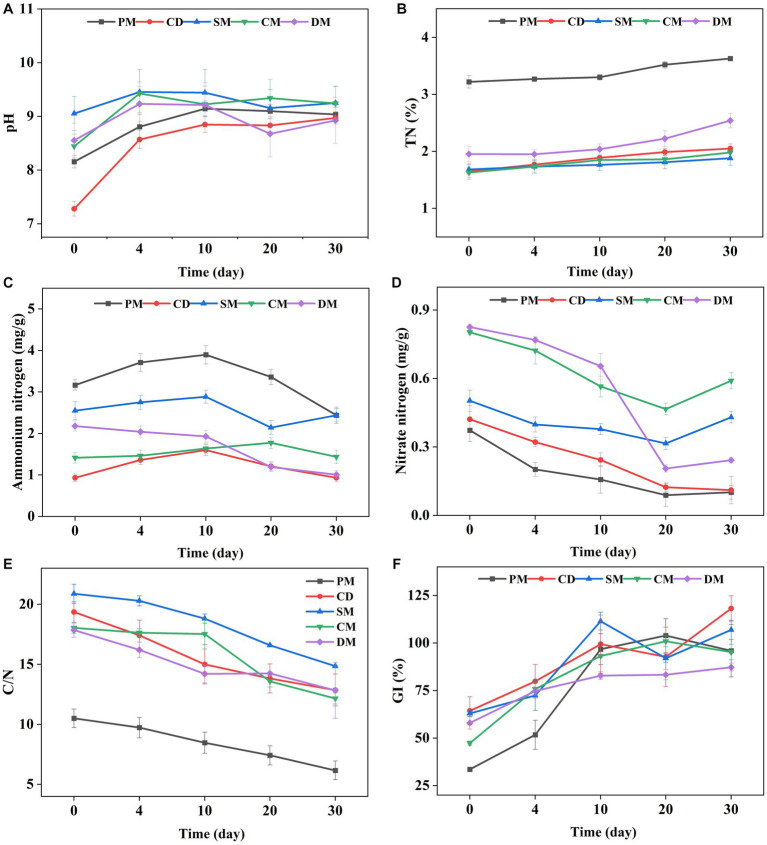
The changes of pH **(A)**, total nitrogen (TN) **(B)**, ammonium nitrogen **(C)**, nitrate nitrogen **(D)**, the ratio of carbon to nitrogen (C/N) **(E)**, and germination index (GI) **(F)**, in manure composting from different sources. PM, pig manure composting; CD, cow dung composting; SM, sheep manure composting; CM, chicken manure composting; DM, duck manure composting.

The change trend of temperature in five treatments was consistent during composting (Supplementary Figure S1). The temperature of PM and CD was significantly higher than that of other treatments at day 17–23. The duration time of temperature > 50°C in the five groups exceeded 7 days, which met the requirement of killing pathogens in composting (Wang et al., 2020). The EC of the five groups all was below 4 ms/cm at the end of composting, meeting the criteria for mature composts (Supplementary Figure S1) (Bernal et al., 2009). The initial pH values of PM, SM, CM, and DM were weakly alkaline, ranging from 8.16 to 9.05, however, CD was close to neutral at 7.28. After the 4th day, the pH of all composts gradually stabilized at about 9 (Figure 1A).

TN of all composts showed an increasing trend (Figure 1B) and the initial TN content of PM was the highest (3.22%). After 30 days of composting, TN of five treatments (PM, CD, SM, CM, and DM) increased by 0.41, 0.40, 0.20, 0.35 and 0.59%, respectively. The increase of TN was related to the greater degradation rate of organic carbon than the loss rate of nitrogen, leading to the concentration of nitrogen (Zheng et al., 2021). At the end of composting, the ammonium nitrogen content of PM, SM and DM decreased by 23.04, 4.36 and 53.87%, respectively, while the ammonium nitrogen content of CD and CM increased by 0.22 and 1.64%, respectively (Figure 1C). It was reported that the high-temperature alkaline environment led more ammonium nitrogen loss by promoting the conversion of NH_4_^+^−N to NH_3_ (Liu et al., 2023). It may be also affected by the rapid growth and propagation of ammonia-oxidizing bacteria to promote the decomposition of nitrogen-containing organics in compost (Wang et al., 2013). The content of nitrate nitrogen in the five treatments was decreased during composting (Figure 1D). The nitrate nitrogen of SM, CM, and DM decreased firstly and then increased at the maturity stage of composting, indicating that the activity of nitrifying bacteria might increase and NH_4_^+^−N was converted to NO_3_^−^−N through nitrification as the decrease of ammonium nitrogen after day 20 (Figure 1C) (Xu et al., 2022).

The C/N related to the decomposition of organic matter by microorganisms and GI reflecting the toxic effect of composts on plants, are always regarded as maturity index for composting (Bernal et al., 2009). The C/N ratio of the five treatments basically showed a downward trend (Figure 1E) and the C/N ratio of PM, CD, SM, CM, and DM decreased by 4.33, 6.49, 6.02, 5.87, and 5.01 at the end of composting compared to the initial stage. The C/N of CD and SM decreased more compared to other groups, which indicated that the organic matter decomposition of cow manure and sheep manure was faster. The GI of all treatments showed an increasing trend (Figure 1F). As for the samples from 0 d of composting, GI decreased as the following: CD > SM > DM > CM > PM, which might be related to the less toxic to seeds of plant-derived waste (cattle and sheep are mainly herbivorous) (Li et al., 2023). After day 10 of composting and until the end of composting, GI in all the treatments was above 70%, suggesting that the compost products were completely decomposed and had no toxicity to plants (Wang et al., 2020). On day 30, GI decreased as the following trend: CD > SM > PM > CM > DM. Compared to the GI of the initial stage, it could be found that PM had a more obvious increase of maturity level compared to other composts during composting.

### 3.2. Organics degradation of manure compost from different sources

**Figure 2:**
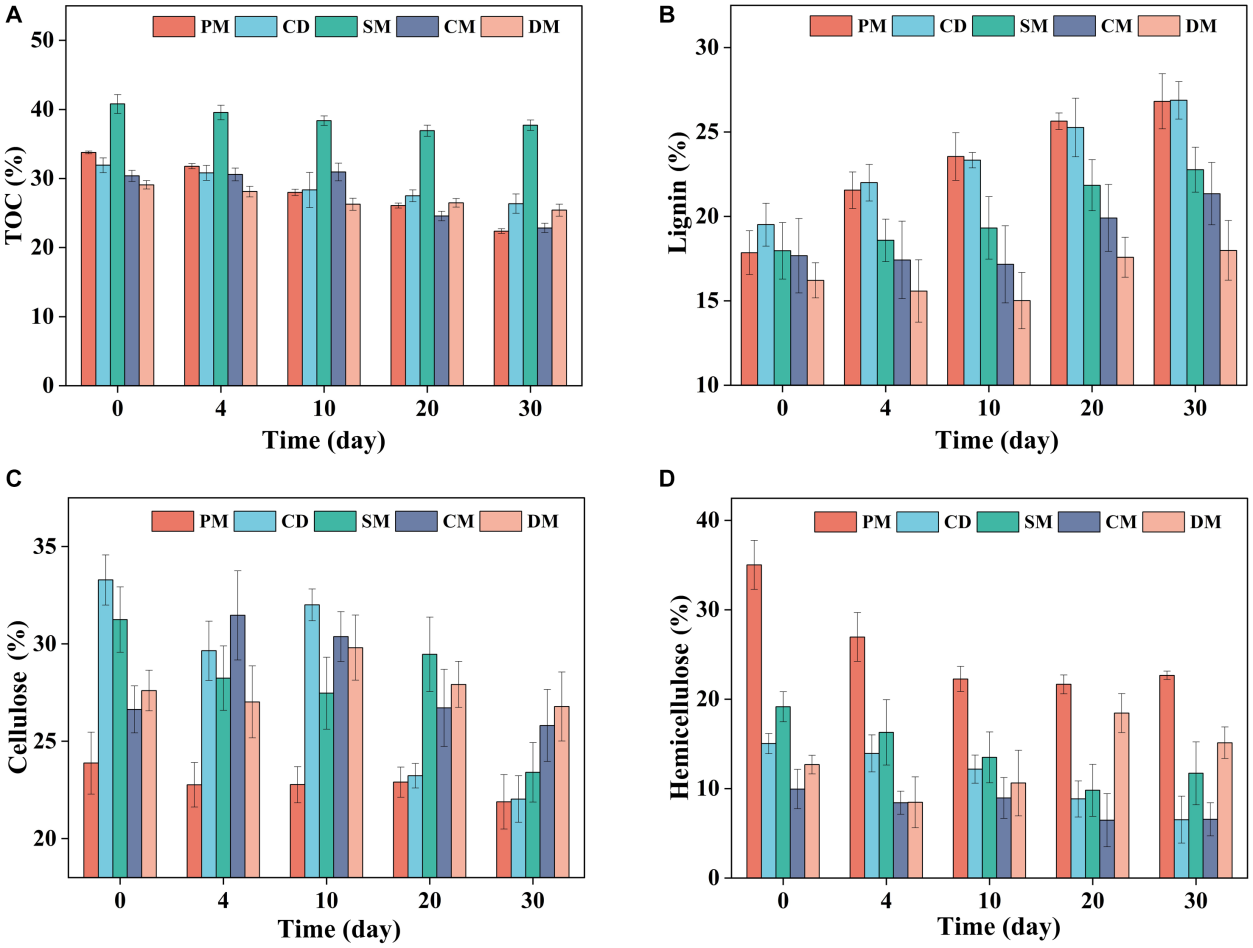
Changes of total organic carbon (TOC) **(A)**, lignin **(B)**, cellulose **(C)**, and hemicellulose **(D)**, content in manure composting from different sources. PM, pig manure composting; CD, cow dung composting; SM, sheep manure composting; CM, chicken manure composting; DM, duck manure composting.

The degradation of total organic matter is always synchronous with the change of TOC, and it can be found that the TOC content of five treatments showed a decreasing trend (Figure 2A). After 30 days composting, the TOC content of PM, CM, SM, CD, and CM decreased by 11.38, 5.56, 3.09, 7.53, and 3.67%, respectively. The organics degradation of PM and CM was higher than other composts, which was related to the rich protein, lipids and low molecular weight organic acids in pig manure and chicken manure (Kong et al., 2022).

Considering that lignocellulosic fractions, as recalcitrant organic fractions, had a high proportion in animal manure, the changes of cellulose, hemicellulose, and lignin in composting of five groups were compared as shown in Figure 2. The ratio of lignin in dry matter was increased during composting due to the concentration effect as the difficulty of lignin degradation (Tuomela et al., 2000). After 30 days of composting, the cellulose content of PM, CD, SM, CM, and DM decreased by 1.99, 11.25, 7.84, 0.83, and 0.82%, respectively, and the degradation of cellulose in CD and SM was significantly higher than other composts (*p* < 0.05) (Figure 2C). Compared with other groups, the cellulose in composting from cow manure and sheep manure was more easily to be degraded. At the end of composting, the hemicellulose content of PM, CD, SM, and CM decreased by 12.35, 8.51, 7.46, and 3.38%, respectively, and the degradation of hemicellulose in PM was significantly higher than other composts (*p* < 0.05), while the ratio of hemicellulose in dry matter of DM increased by 2.46% (Figure 2D). Ghanney et al. (2023) found that when the initial content of hemicellulose in the compost was higher than that of cellulose, hemicellulose was more likely to be degraded than cellulose and lignin. The above results showed that the degradation of cellulose in composting from cow manure and sheep manure was obvious and the degradation of hemicellulose in composting from pig manure and chicken manure was prominent, indicating that the degradation of organic components might be related to its initial content.

### 3.3. Changes of humification index of manure compost from different sources

**Figure 3:**
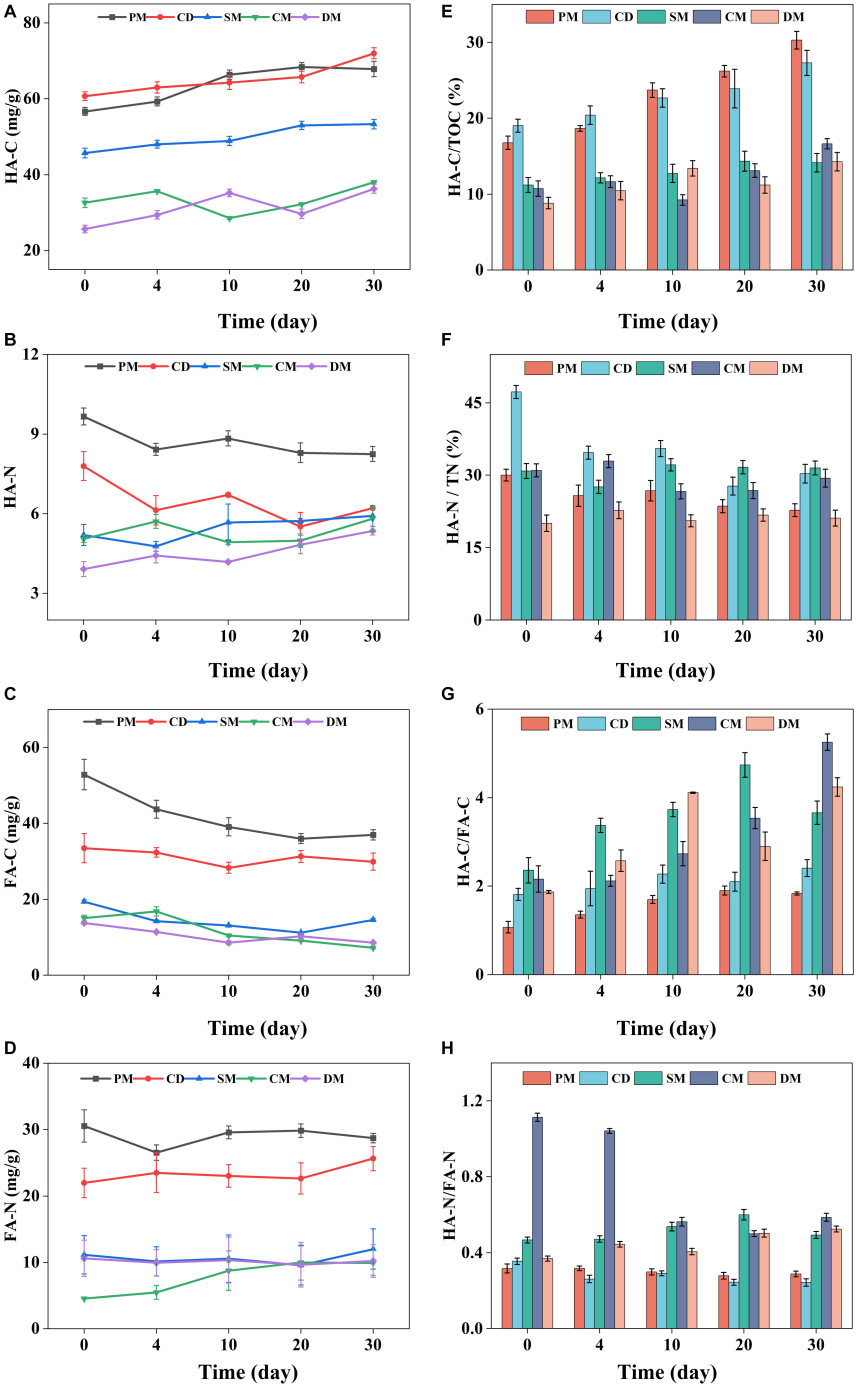
Changes of HA-C **(A)**, HA-N **(B)**, FA-C **(C)**, FA-N **(D)**, HAC/TOC **(E)**, HA-N/TN **(F)**, HA-C/FA-C **(G)**, and HA-N/FA-N **(H)**, during manure composting from different sources. HA, humic acid; FA, fulvic acid; C, carbon; N, nitrogen; TOC, total organic carbon. PM, pig manure composting; CD, cow dung composting; SM, sheep manure composting; CM, chicken manure composting; DM, duck manure composting.

The content of HA and FA based on carbon level were analyzed to compare the humification process and humification index of manure composting from five sources, and nitrogen content of HA and FA was also investigated. Based on carbon (C) element, the HA-C content of PM, CD, and SM showed a gradual upward trend during composting and the HA-C of CM and DM was fluctuant (Figure 3A). The slight decrease of HA-C during composting may be related to the decomposition of simple components in HA-C by microorganism (Wei et al., 2007). As FA, an unstable humus fraction, is easy to be decomposed and utilized by microorganism (Wang et al., 2022), the C content of FA in all manure composting decreased obviously (Figure 3B). The C content of HA and FA in composting from pig manure and cow dung was significantly higher than that in other manure composts, similar to previous reports (Liu et al., 2022). The HA-C/TOC and HA-C/FA-C indices can reflect the maturity and humification degree of composts and the conversion of organic matter to humus (Kong et al., 2022). The HA-C/TOC of each treatment basically showed an increasing trend (Figure 3E). During composting, the HA-C/TOC of PM and CD was higher than that of other treatments, while the HA-C/TOC of SM had the least increase after composting. This was attributed to the fact that TOC of PM and CD might be easier to be broken down and converted into HA-C, and SM was rich in lignocellulose with a lower mineralization rate of organic matter (Li et al., 2023). In addition, at the end of composting, the HA-C/FA-C of five treatments increased by 0.59 ~ 3.09 (Figure 3G), indicating that the structure of humus became more complex at the end of aerobic composting, and this change was particularly obvious in the compost of sheep manure, chicken manure and duck manure.

The change of nitrogen (*N*) content of HA among the five treatments was different (Figure 3B). The HA-N of PM and CD decreased firstly and then stabilized. However, the HA-N of SM, CM and DM showed a floating upward trend. Compared with day 0, the HA-N of PM and CD at the end of composting decreased by 14.62 and 20.29%, respectively, which was mainly decomposed at the early stage of composting. The HA-N of SM, CM and DM increased by 13.84–36.76% after composting and the HA-N content of DM increased more than other groups, suggesting that more nitrogen-containing precursors such as amino acids were involved in the formation of HA in DM composting (Zhang et al., 2018). The *N* in FA was ranged from 5.0 mg/g to 30.2 mg/g in diverse manure composting, which was relative stable in composting except CM with a significant increase (Figure 3D). At the end of composting, HA-N/TN decreased by 24.26, 35.85, and 5.27% for PM, CD, and CM, respectively, and HA-N/FA-N also decreased during composting (Figures 3F,H), while there was no significant change in HA-N/TN and HA-N/FA-N of SM and DM. Besides, the C/N in HA was higher than that in FA in different manure composting, suggesting that FA might be seen as the carrier for *N* to enhance the *N* reservation in composting (Ma et al., 2022). Based on the above results, it was found that *N* of humus in composting of pig manure, cow manure and chicken manure was easy to be transformed and utilized by microorganisms (Wei et al., 2007), but *N* of humus in SM and DM might be used as a potential *N* pool for reducing *N* loss as the humification process of composting.

Based on the three-dimensional fluorescence spectroscopy, HA was divided into four parallel factor components (C1 ~ C4, with progressively more complex structures), and FA was divided into three parallel factor components (C1 ~ C3) (Supplementary Figure S2). In the distribution of HA fractions, PM, CD and SM had lower C1 fractions of 4.16–14.89%, while CM and DM had higher C1 fractions of 16.25–20.92%. DM had the largest decrease in C1 of 9.10%, followed by SM (7.52%). Notably, SM had the greatest increase in C4 fraction with 18.75% compared to other groups, suggesting the more stable and complex HA in SM (Wei et al., 2014). In the distribution of FA parallel factor components, the C1 component of FA in PM had a small percentage, lower than C2 and C3. The proportions of C1, C2 and C3 fractions in CD, SM, CM, and DM were close to each other. The C3 fraction increased during composting of diverse groups, and DM had the largest increase in the proportion of C3, followed by CM. Combined with the HAC/FAC indicators, the above results indicates that SM, and DM were more inclined to form HA with more complex structure.

### 3.4. Influencing factors of humification process of different manure composts

In order to clarify the humification characteristics of different livestock and poultry manure composts, cluster analysis was carried out based on GI, C/N, HA-C, HA-C/TOC, and HA-C/FA-C (Supplementary Figure S3). Two categories were found and the manure composts in same category had similar humification process and the degree of maturity (Wei et al., 2022). Category A mainly included PM, CD, and category B mainly included SM, CM, and DM. Based on the previous results, it could be found that the category A had more HA and FA in composting compared to category B.

**Figure 4:**
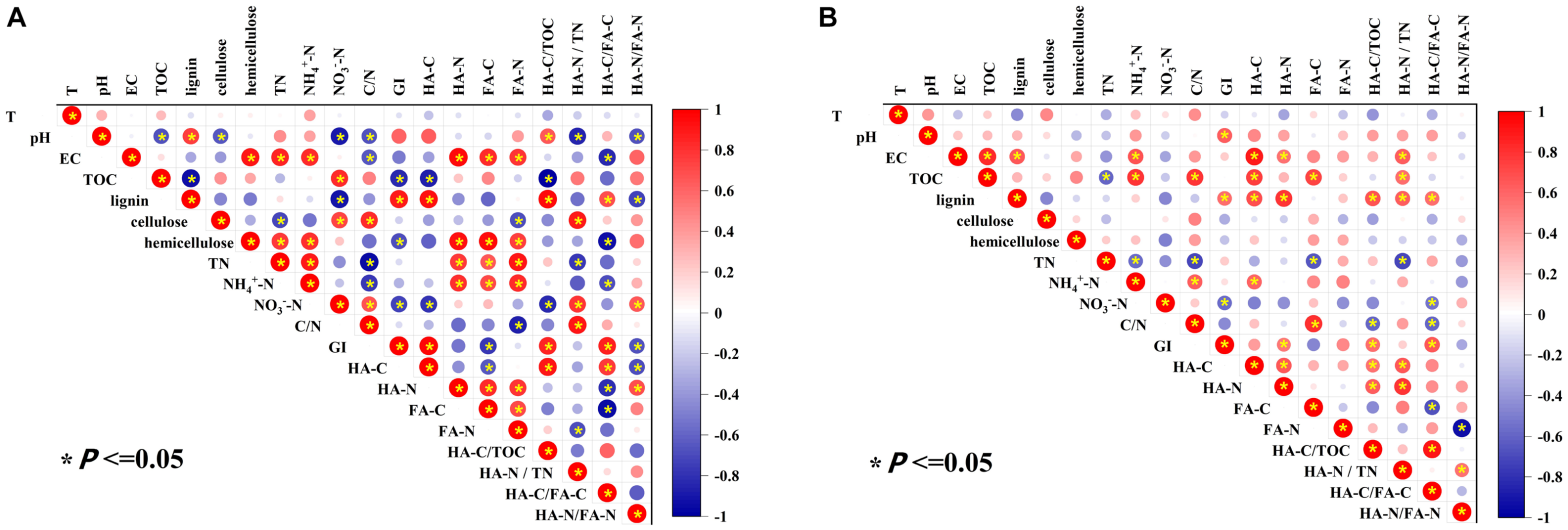
Pearson correlation analysis of basic physicochemical and humification indexes of manure composting [**(A)** Category A including composts from pig manure and cow dung; **(B)** Category B including composts from sheep manure, chicken manure, and duck manure compost].

Pearson’s correlation analysis was performed to detect the influencing factors of maturity and humification indicators in the two major categories (A, B) of manure compost from different sources (Figure 4). In category A, pH showed a significantly positive correlation with HA-C/TOC (*p* < 0.05) and a significant negative correlation with HA-N/TN and HA-N/FA-N (*p* < 0.05), indicating that the increase of pH was favorable to the conversion of organic C fractions to HA-C in composting from pig manure and cow dung and would inhibit the conversion of *N* fractions toward HA-N with complex structure. Hemicellulose was significantly negatively correlated with GI, HA-C/FA-C (*p* < 0.05), which indicated that hemicellulose decomposition might produce small-molecule precursors of HA, promoting the formation of HA with more complex structure (Zhao et al., 2022).

In class B, the effect of pH on humification process of composting was the same as that of class A. There was a significantly positive correlation between lignin and HA-C, HA-N, and HA-C/TOC (*p* < 0.05), indicating that the enrichment of lignin in manure belonging to the class B was conducive to the formation of HA in composting. There was a significant negative correlation between nitrate and HA-C/FA-C (*p* < 0.05). These different key influencing factors may help to regulate the content of humus in composting from different sources.

### 3.5. Changes in microbial community composition and diversity

**Figure 5:**
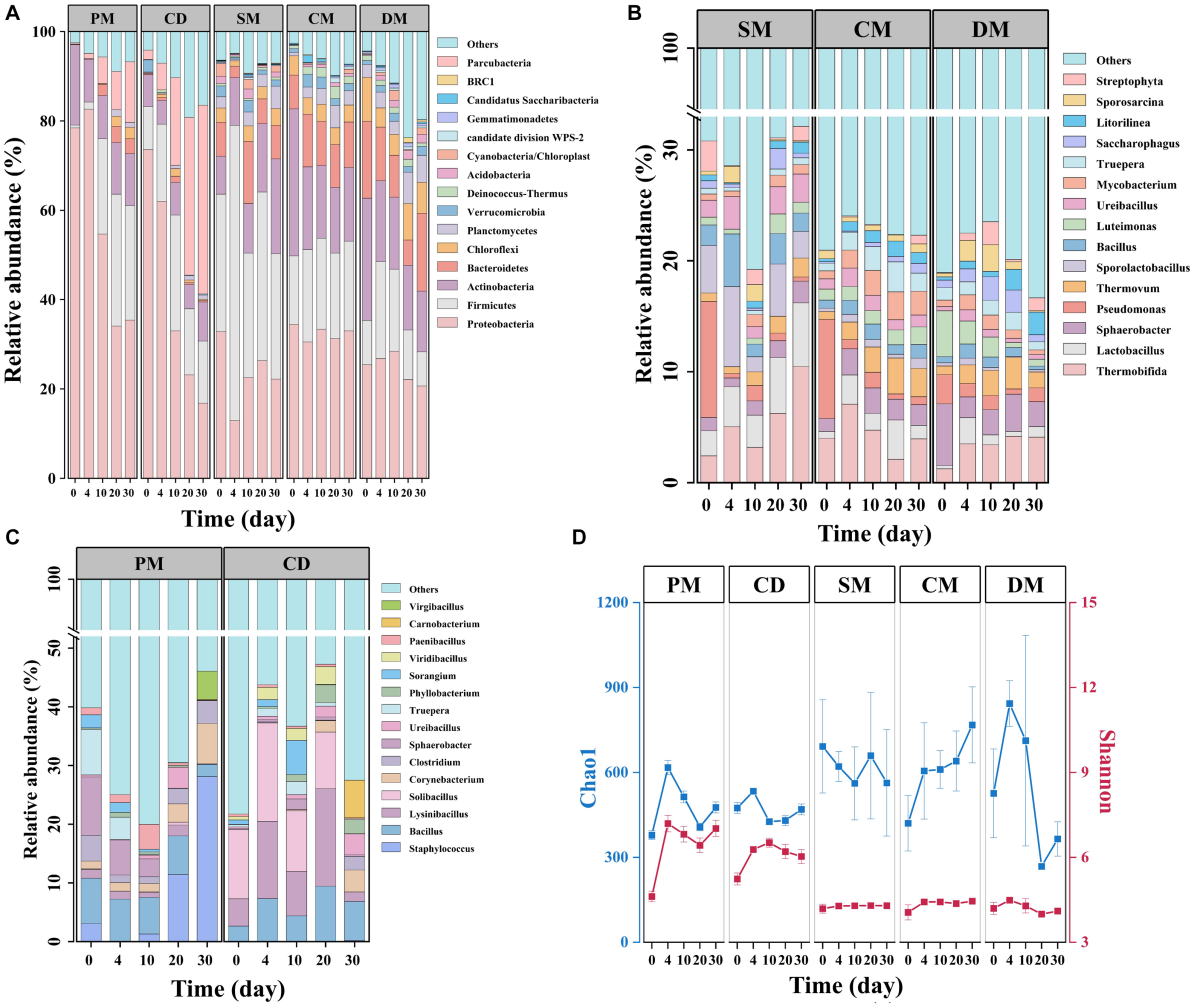
Bacterial community succession and diversity in manure composts from different sources. **(A)** Phylum-level bacterial community composition of five manure composts; **(B)** Genus-level bacterial community composition of manure composts from Category A (PM, CD); **(C)** Genus-level bacterial community composition of manure composts from Category B (SM, CM, DM); **(D)** α-diversity index (Shannon’s index and Chao1). PM, pig manure composting; CD, cow dung composting; SM, sheep manure composting; CM, chicken manure composting; DM, duck manure composting.

At the bacterial phylum level (Figure 5A), Proteobacteria was the most abundant in PM and CD, accounting for 76.0–79.5% of the total phyla, followed by Actinobacteria (18.4%) and Firmicutes (10.0%), respectively. During the thermophilic stage, the bacterial community structure in PM and CD changed significantly, with an increase in the relative abundance of Firmicutes and Parcubacteria and a significant decrease in the relative abundance of Proteobacteria, similar to previous reports (Zhang et al., 2021). In SM, Firmicutes replaced the dominance of *Proteobacteria* (27.9–66.1%) after the thermophilic phase, which may be due to the high cellulose content of sheep manure and the important role of Firmicutes in cellulose decomposition (Yang et al., 2023). In addition, Proteobacteria, Firmicutes, Actinobacteria, Bacteroidetes, and Chloroflexi occupied more than 60% of the total bacterial phyla in chicken and duck manure composting, which is in line with previous reports in poultry manure compost (Zhang et al., 2018). Proteobacteria was reported to be thermotolerant and involved in carbon cycling and nitrogen transformation, and Chloroflexi was associated with amino acid and carbohydrate metabolism (Ma et al., 2022), which might be one of the main phyla during the thermophilic and maturity stages of composting to promote the humification process.

At the genus level, the composition of the top 15 genera differed significantly between the two major categories of manure composting (Figures 5B,C). In the composting from category A, *Staphylococcus* sp. dominated (12.3–38.0%) in the cooling period (day 20–30) of PM. *Bacillus* sp., *Lysinibacillus* sp. and *Solibacillus* sp. were the dominant genera in CD during day 0–20 of composting. The relative abundance of *Carnobacterium* sp. that could decompose carbohydrates such as cellobiose at low temperatures increased sharply on the maturity stage of composting. Among the three composts in the major category, *Pseudomonas* sp. was the dominant genus in SM and CM (9.0 to 10.44%). *Thermobifida* was dominant in all three manure composts of group B during the high temperature and maturity stages. It was reported that *Thermobifida* sp. belonged to the group of thermophilic actinomycetes, which produced large amounts of cellulose degrading enzymes (Kukolya et al., 2002).

The changes of alpha diversity index Chao1 and Shannon were similar for PM and CD treatments within 30 d of composting (Figure 5D). Except for the sheep manure compost, the Chao1 index of the other four treatments increased on day 4, which was attributed to the decomposition of abundant organic matter that could be directly utilized by microorganisms. The Chao1 indices of PM and DM started to decrease after day 4, which may be due to the high temperature inhibition (Liu et al., 2020). The Shannon indices of all the composts in the major category B were smaller than those of the A major category. Therefore, the microbial composition in different manure composting varied, and the dominant microorganisms continued to change during composting, which might reshape composting microbiome and their interactions, ultimately altering the microbial communities’ function.

### 3.6. Effect of microbial community on humification of manure composting

**Figure 6:**
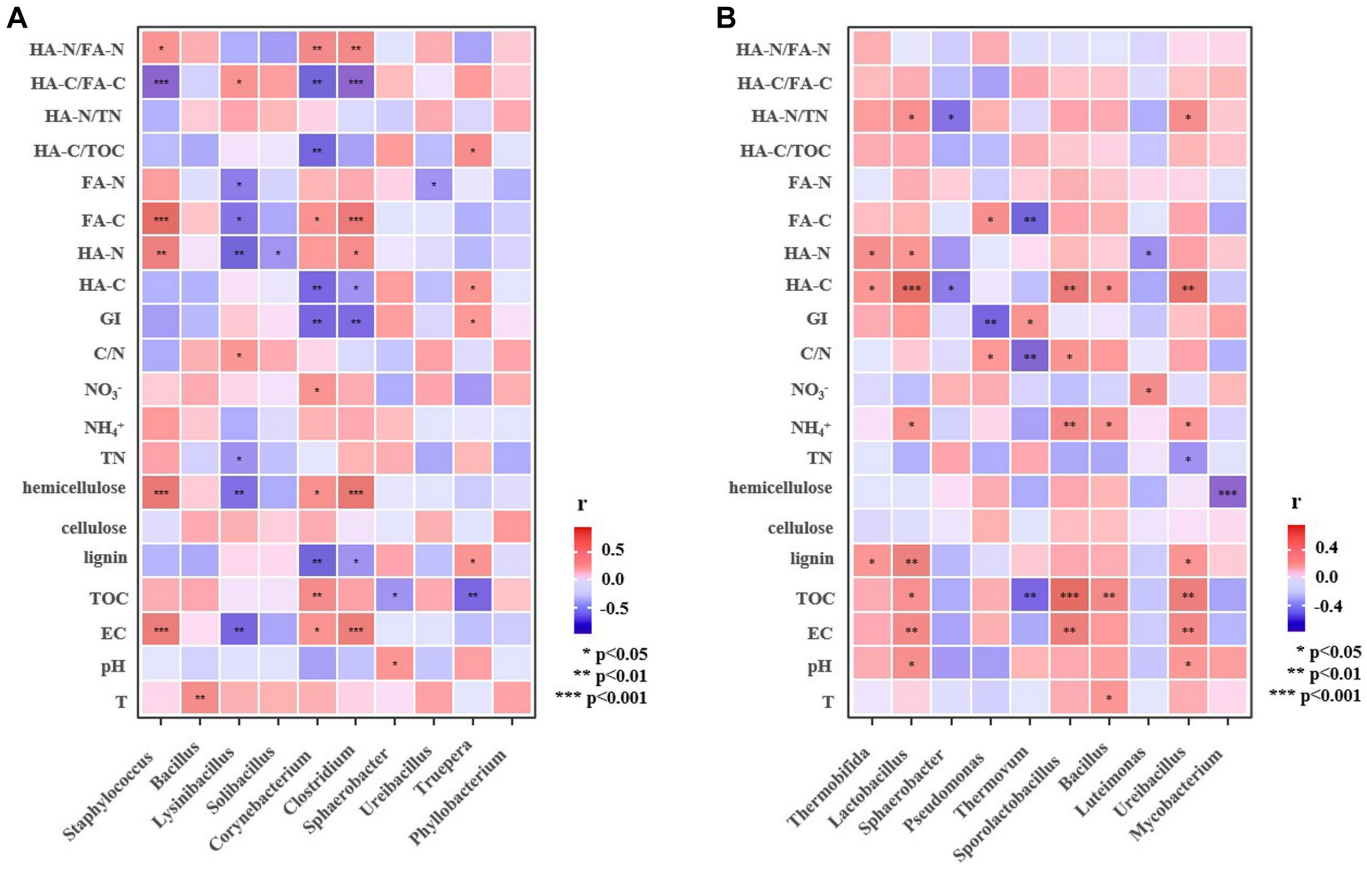
Pearson correlation analysis of dominant bacterial genera and different factors related to humification (a, Category [**(A)** including composts from pig manure and cow dung; b, Category [**(B)**, including composts from sheep manure, chicken manure, and duck manure).

To further explore the potential roles of microbial community, Pearson correlation analysis was performed for the top 10 dominant bacterial genera and physicochemical and humification indexes. In manure composting of category A, *Truepera* was negatively correlated with TOC (*p* < 0.05), but positively correlated (*p* < 0.05) with GI, HA-C and HA-C/TOC (Figure 6A). This suggested that *Truepera* could promote the mineralization of organic matter and convert it to humic acid (Wang et al., 2021). *Lysinibacillus*, as the dominant genus of compost category A, had a significant negative correlation (*p* < 0.05) with hemicellulose, FA-C, and HA-N, and a significant positive correlation (*p* < 0.05) with HA-C/FA-C, indicating that *Lysinibacillus* might benefit the decomposition of hemicellulose to small molecules FA. It was reported that *Lysinibacillus* (*Firmiculus*) was usually found in the high temperature phase of composting with the function of the degradation of dissolved organic matter (Liu et al., 2020), which could improve the humification degree and aromaticity of humus (Ma et al., 2022). In composts belonging to category B, *Thermovum* was significantly negatively correlated with TOC, FA-C and C/N (*p* < 0.01), but positively correlated (*p* < 0.05) with GI (Figure 6B). In the high temperature phase, as a thermophilic bacterium, *Thermovum* can promote the degradation of organic matter in compostin (Zhang et al., 2018). Moreover, *Thermobifida*, *Lactobacillus* and *Ureibacillus* were significantly (*p* < 0.05) or extremely significantly (*p* < 0.01) positively correlated with lignin and HA-C, demonstrating that the presence of these three bacteria contributed to the formation of lignin-derived humus in the manure composting from group B. It has been reported that peroxidase secreted by *Thermobifida* can act synergistically with xylanase on lignocellulosic degradation to produce aromatic compounds, aldehydes and organic acids, which may sustain the growth of other microorganisms or directly involved in the formation of HA (Reshmy et al., 2022).

**Figure 7:**
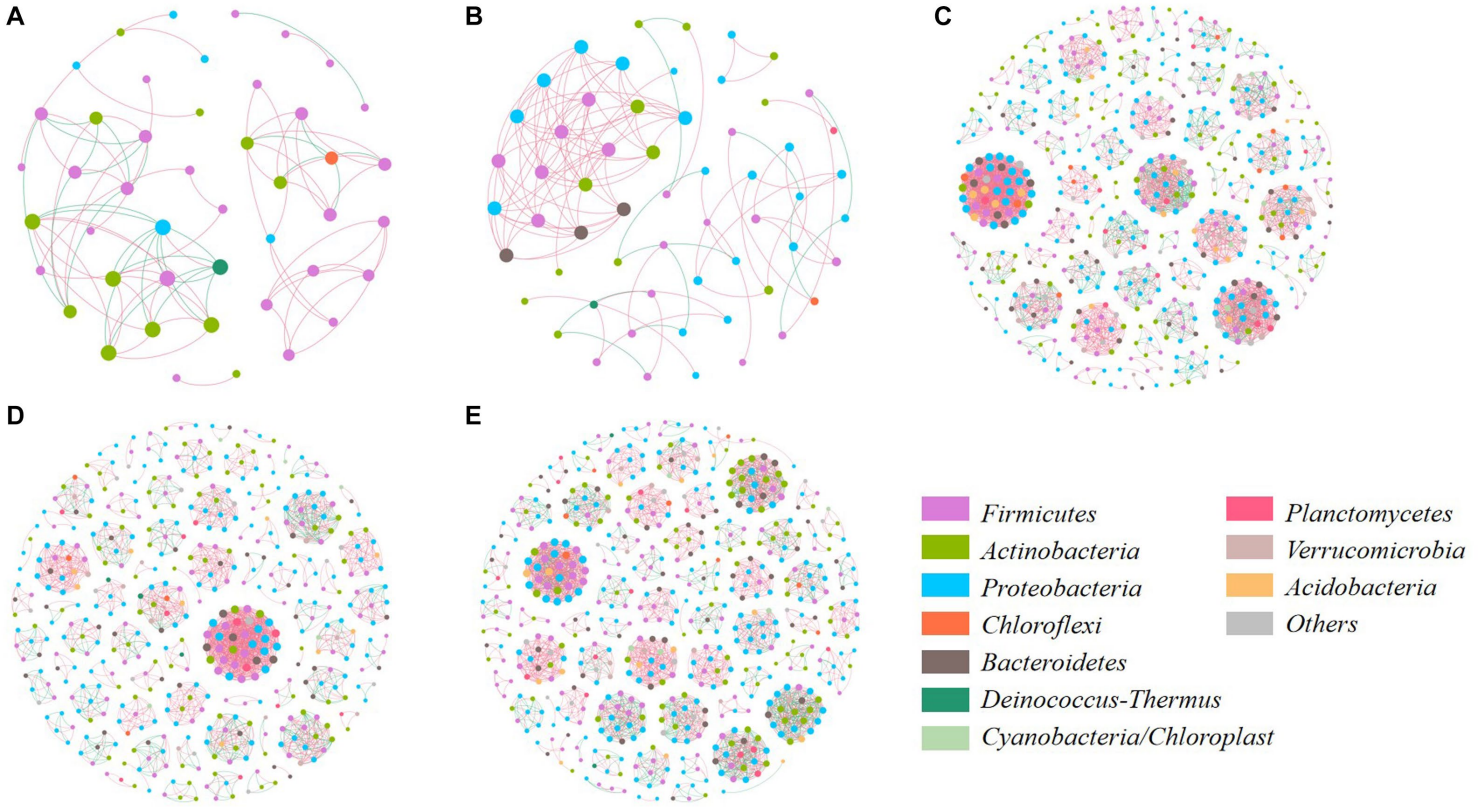
Network analysis of microbial interaction at genus level. **(A)** PM; **(B)** CD; **(C)** SM; **(D)** CM; **(E)** DM. The node size indicates the abundance of microbes. The node color indicates different phyla. The blue and red lines represent positive and negative correlations, respectively. PM, pig manure composting; CD, cow dung composting; SM, sheep manure composting; CM, chicken manure composting; DM, duck manure composting.

Co-occurrence network was further used for analyzing the relationship of core bacterial communities and their interaction in manure composts from different sources (Figure 7; Supplementary Table S2). The topological structure of bacterial OTUs had an obvious difference between the category A (PM and CD) and B (SM, CM, and DM). The number of core bacterial nodes in the network of category A was significantly lower than that in category B. The more complex network occurred in SM, CM, DM, which contained more links, and modules with a higher clustering coefficient. Therefore, it could be found that the ecological interaction patterns of bacterial communities in composting are sensitive to the sources of manure, and the composts with higher humification indices and humus content would reflect directly on the bacterial interaction.

## 4. Conclusion

The decomposition of cellulose in CD and SM was higher in composting, and the hemicellulose degradation rate in PM and CM was higher than other manure composting. The content of humus components of PM and CD was significantly higher than that of other composts. More HA with complex structure was formed in SM composting, while the formed FA in CM and CM tended to be more complex. Pearson correlation analysis showed that the humification process in PM and CD (category A) was influenced by the formation of lignin precursors and hemicellulose degradation, while humification process in SM, CM, DM (category B) was mainly restricted by the decomposition rate of organics and lignin. Key bacteria *Lysinibacillus* were identified to promote the degradation of hemicellulose and the conversion of FA-C to HA-C in composting from category A, and *Thermobifida*, *Lactobacillus* and *Ureibacillus* that significantly positively correlated with lignin and HA-C were the key bacteria in composting of category B. The interaction patterns of core bacterial communities in manure composting were sensitive to the sources of raw materials, reflecting the humification indices and humus content (humification process) of composting.

## Data availability statement

The raw data supporting the conclusions of this article will be made available by the authors, without undue reservation.

## Author contributions

YL: Data curation, Formal analysis, Writing – original draft. JL: Data curation, Methodology, Writing – original draft. YC: Methodology, Software, Writing – original draft. RL: Data curation, Formal analysis, Software, Writing – original draft. KZ: Formal analysis, Software, Writing – original draft. YZ: Conceptualization, Data curation, Formal analysis, Investigation, Methodology, Resources, Validation, Visualization, Writing – original draft. RW: Data curation, Visualization, Writing – original draft. YW: Conceptualization, Formal analysis, Funding acquisition, Project administration, Resources, Supervision, Validation, Writing – review & editing.
