# Supplementary material for: Comparing bacterial dynamics for the conversion of organics and humus components during manure composting from different sources
Source: Front Microbiol. 2023 Sep 28;14:1281633. doi: 10.3389/fmicb.2023.1281633 (PMC10568323; doi:10.3389/fmicb.2023.1281633)
Supplement: Supplementary file 1 [file Data_Sheet_1.pdf]

Supplementary Material

Comparing bacterial dynamics for the conversion of organics and humus components during manure composting from different sources

Yan Li[1,2,#], Jun Li[3,4,#], Yuan Chang[3,4], Ruoqi Li[3,4], Kaiyun Zhou[3,4], Yabin Zhan[3,4,5,*], Renyue Wei[6], Yuquan Wei[3,4,*]

[1] *Department of Center Laboratory, Haikou Municipal People's Hospital and Central South University Xiangya Medical College Affiliated Hospital, Haikou 570208, Hainan, China*

[2] *Key Laboratory of Tropical Biological Resources, Ministry of Education; Key Laboratory for Marine Drugs of Haikou, Hainan University, Haikou 570228, China*

[3] *College of Resources and Environmental Science, Beijing Key Laboratory of Biodiversity and Organic Farming, China Agricultural University, 100193, Beijing, China*

[4] *Organic Recycling Institute (Suzhou) of China Agricultural University, Wuzhong District, Suzhou 215128, China*

[5] *Key Laboratory of Fertilization from Agricultural Wastes, Ministry of Agriculture and Rural Affairs, Institute of Plant Protection and Soil Fertilizer, Hubei Academy of Agricultural Sciences, Wuhan, Hubei 430064, China*

[6] *College of Life Science, Northeast Agricultural University, Harbin 150030, China*

---

[*] Corresponding author

E-mail addresses: zhanyabin@126.com (Y. Zhan); weiyq2013@gmail.com (Y. Wei)

[#] Authors contributed equally to this work.

Table S1. Basic properties of different raw materials

Ingredients	Moisture content[a]	pH[a]	TOC(%)[b]	TN(%)[b]	C/N[b]
Pig manure	69.70±1.67	8.13±0.01	26.28±0.87	3.83±0.11	6.86±0.08
Cow dung	65.61±1.72	7.91±0.03	22.35±0.64	1.84±0.06	12.15±0.25
Sheep manure	54.83±0.68	9.17±0.18	35.27±2.24	2.24±0.06	15.75±0.43
Chicken manure	51.92±2.50	8.55±0.18	26.57±1.41	3.04±0.01	8.75±0.15
Duck manure	60.82±1.67	8.68±0.21	25.03±2.25	2.25±0.02	11.27±0.21
Sawdust	8.31±0.17	5.61±0.01	46.02±0.02	0.26±0.02	175.51±0.47

a: Calculated on wet basis; b: Calculated on dry basis.

TOC: total organic carbon, TN: total nitrogen, C/N: the ratio of carbon to nitrogen.

Table S2 Topological properties of the bacterial community ecological networks in different treatments.

Treatments	Network size[a]	Total link[b]	avgK	HD	Modularity
PM	42	64	2.091	1.011	0.037
CD	55	87	2.128	1.207	0.056
SM	428	1020	6.681	6.114	0.467
CM	488	942	6.663	6.320	0.436
DM	515	1150	6.702	6.891	0.472

[a] Number of nodes detected in the network.

[b] Total links in the ecological network included both positive and negative links.

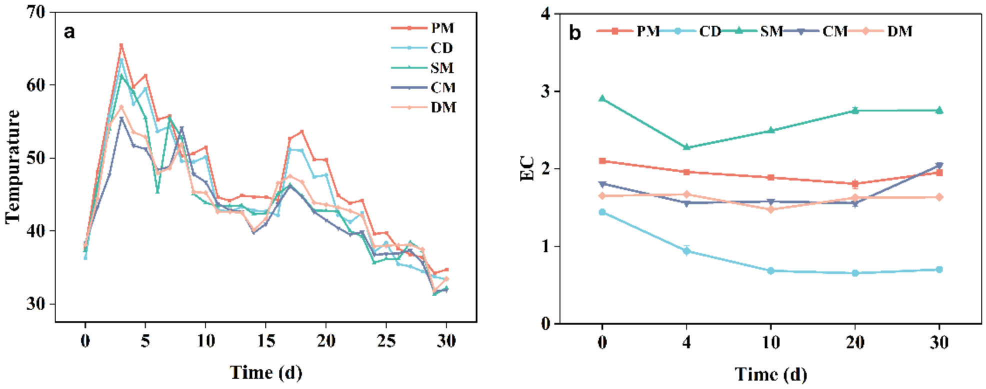

Fig. S1. The changes of temperature (a) and electrical conductivity (EC) (b) in manure composting from different sources. PM, pig manure composting; CD, cow dung composting; SM, sheep manure composting; CM, chicken manure composting; DM, duck manure composting.

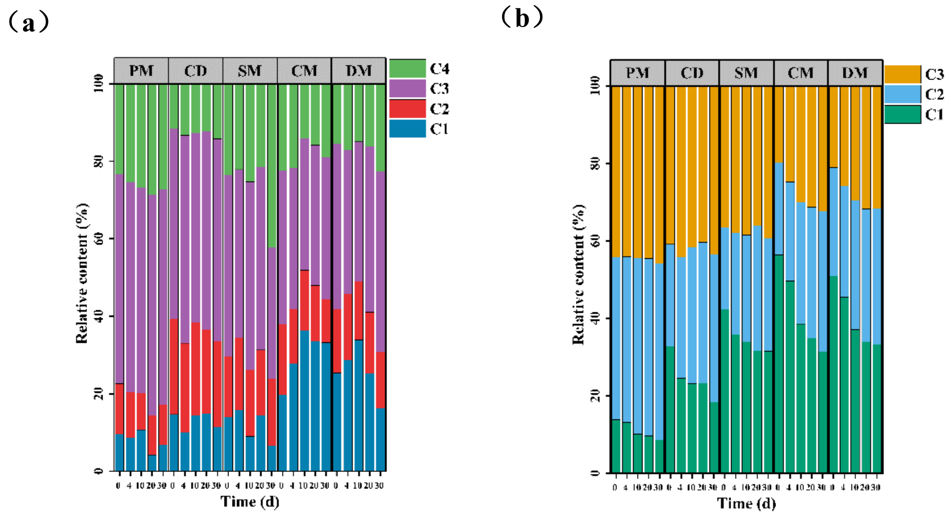

Fig. S2. Distribution of parallel factor components of humus (a, humic acid; b, fulvic acid) in different composts. PM, pig manure composting; CD, cow dung composting; SM, sheep manure composting; CM, chicken manure composting; DM, duck manure composting.

.

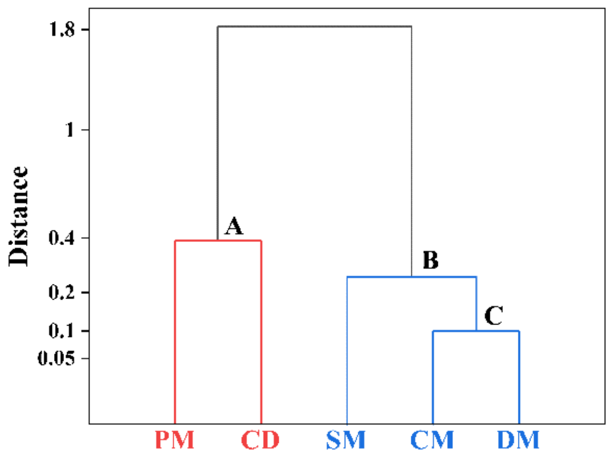

Fig. S3. Cluster analysis of manure composts from different sources according to organics decomposition and humification index. PM, pig manure composting; CD, cow dung composting; SM, sheep manure composting; CM, chicken manure composting; DM, duck manure composting.
